# Phenolic Acid Composition, Antiatherogenic and Anticancer Potential of Honeys Derived from Various Regions in Greece

**DOI:** 10.1371/journal.pone.0094860

**Published:** 2014-04-21

**Authors:** Eliana Spilioti, Mari Jaakkola, Tiina Tolonen, Maija Lipponen, Vesa Virtanen, Ioanna Chinou, Eva Kassi, Sofia Karabournioti, Paraskevi Moutsatsou

**Affiliations:** 1 Department of Biological Chemistry, Medical School, University of Athens, Athens, Greece; 2 CEMIS-Oulu, Kajaani University Consortium, University of Oulu, Sotkamo, Finland; 3 Laboratory of Pharmacognosy and Chemistry of Natural Products, Department of Pharmacy, University of Athens, Panepistimioupolis, Athens, Greece; University of Catania, Italy

## Abstract

The phenolic acid profile of honey depends greatly on its botanical and geographical origin. In this study, we carried out a quantitative analysis of phenolic acids in the ethyl acetate extract of 12 honeys collected from various regions in Greece. Our findings indicate that protocatechuic acid, p-hydroxybenzoic acid, vanillic acid, caffeic acid and p-coumaric acid are the major phenolic acids of the honeys examined. Conifer tree honey (from pine and fir) contained significantly higher concentrations of protocatechuic and caffeic acid (mean: 6640 and 397 µg/kg honey respectively) than thyme and citrus honey (mean of protocatechuic and caffeic acid: 437.6 and 116 µg/kg honey respectively). p-Hydroxybenzoic acid was the dominant compound in thyme honeys (mean: 1252.5 µg/kg honey). We further examined the antioxidant potential (ORAC assay) of the extracts, their ability to influence viability of prostate cancer (PC-3) and breast cancer (MCF-7) cells as well as their lowering effect on TNF- α-induced adhesion molecule expression in endothelial cells (HAEC). ORAC values of Greek honeys ranged from 415 to 2129 µmol Trolox equivalent/kg honey and correlated significantly with their content in protocatechuic acid (p<0.001), p-hydroxybenzoic acid (p<0.01), vanillic acid (p<0.05), caffeic acid (p<0.01), p-coumaric acid (p<0.001) and their total phenolic content (p<0.001). Honey extracts reduced significantly the viability of PC-3 and MCF-7 cells as well as the expression of adhesion molecules in HAEC. Importantly, vanillic acid content correlated significantly with anticancer activity in PC-3 and MCF-7 cells (p<0.01, p<0.05 respectively). Protocatechuic acid, vanillic acid and total phenolic content correlated significantly with the inhibition of VCAM-1 expression (p<0.05, p<0.05 and p<0.01 respectively). In conclusion, Greek honeys are rich in phenolic acids, in particular protocatechuic and p-hydroxybenzoic acid and exhibit significant antioxidant, anticancer and antiatherogenic activities which may be attributed, at least in part, to their phenolic acid content.

## Introduction

Honey is a highly nutritious natural food product which has been used in various medicinal traditions throughout the world for its healing, antibacterial and antiinflammatory properties. Emerging evidence suggests that honey possesses chemopreventive, antiatherogenic and immunoregulatory properties as well as a great potential to serve as a natural food antioxidant [1]–[7].

Characterization of components in honey that might be responsible for its biological properties is of great interest. Honey contains about 200 substances including sugars, phenolic acids, flavonoids, amino acids, proteins, vitamins and enzymes [8]. Phenolic compounds are considered among the main constituents contributing to the antioxidant and other beneficial properties of honey [9]–[13]. Phenolic acid profile has been determined in various honeys and is considered as a useful tool for determination of the floral origin of honey. Phenolic acids like caffeic acid and p-coumaric acid in chestnut honey as well as protocatechuic acid in honeydew honeys have been used as floral markers [14], [15]. Phenolic acids are compounds with multiple biological activities, including anticancer, antiinflammatory, antioxidant and antiatherogenic properties. Hydroxybenzoic acid derivatives like p-hydroxybenzoic, protocatechuic and vanillic acid as well as hydroxycinnamic acid forms like p-coumaric and caffeic acid, are components with important anticancer activity [16], [17]. Interestingly, protocatechuic and caffeic acid have been also shown to exhibit a significant potential as antidiabetic and cardioprotective agents [18]–[20].

Greece is one of the main producing countries of honey within the EU. In our previous study, we determined the total phenolic content and phenolic acid profile (qualitative analysis) in three Greek honeys [21]. In this study, we carried out a quantitative analysis of phenolic acids in 12 honeys from different collection regions in Greece. Furthermore we evaluated a) their antioxidant potential as oxygen radical absorbance capacity (ORAC), b) their antiinflammatory activity in reducing the expression of adhesion molecules VCAM-1 and ICAM-1 in endothelial cells and c) their ability to influence cell viability of prostate cancer (PC-3) and breast cancer (MCF-7) cells. We also examined possible associations between the composition of honeys in phenolic acids and total phenolic content with their antioxidant, anticancer and antiatherogenic activity.

## Materials and Methods

### Honey Samples

Twelve honey samples harvested from different regions in Greece were obtained and used in this study. These include four samples from commercial honeys (H1–H4) and eight honey samples of different botanical origin (H5–H12) which were provided by certified Greek bee-keepers. Pollen analysis through microscopic examination (see Table S1), characterized the honey samples as follows: four thyme honeys with pollen grains of *Thymus capitatus* in the range of 35% to 62%, six conifer honeys (fir and pine), one honey comprised of a mixture of wildflowers, forest and thyme and one honey from citrus. The honey floral sources and regions of collection are given in Table 1.

**Table 1:** Floral source and regions of honey collection.

Sample Code	Floral Source	Localization
H1	Thyme Attiki (commercial)	Various Greek islands
H2	Wild flowers-forest-thyme Attiki (commercial)	Various Greek regions
H3	Forest Fino (commercial)	Various Greek regions
H4	Fir Attiki (commercial)	Various Greek regions
H5	Thyme 45%^a^	Chania, Crete
H6	Pine	Euboea
H7	Fir	Menalon
H8	Thyme 62%^a^	Iraklio, Crete
H9	Fir	Karpenisi
H10	Pine	Chalkidiki
H11	Thyme 55%^a^	Chania, Crete
H12	Citrus	Argos

a Indicating pollen grains of *Thymus capitatus.*

### Extraction

Each honey sample (3 kg) was diluted with water (1 L). The solution was extracted three times with butanol (3×1 L) and butanol was collected and evaporated to dryness. In order to eliminate the sugar content, it was further extracted with ethyl acetate (3×1 L). The ethyl acetate phase was collected and evaporated to dryness. Subsamples (0.5 g) of dried honey extracts were dissolved in 50% methanol in a volumetric flask (5 ml) and further diluted as needed prior to analysis of total phenolic content, phenolic acids, hydroxy methyl furfural, sugars and oxygen radical absorbance capacity as described below. Subsamples of dried ethyl acetate honey extracts were diluted in DMSO prior to cell culture analysis.

### Chemicals and Reagents

All cell-culture materials, such as HBSS (Hanks balanced salt solution), trypsin-EDTA solution, Dulbecco’s modified essential medium and FBS were obtained from Invitrogen Life Technologies (Carlsbad, CA, USA). TNF-α (T0157), α-Tocotrienol (07205), 3-(4,5-dimethylthiazol-2-yl)-2,5-diphenyltetrazolium bromide (MTT; M5655) and the peroxidase substrate o-phenylendiamine hydrochloride (FASTe OPD; P9187) were obtained from Sigma-Aldrich (St Louis, MO, USA). VCAM-1 antibody (BBA5) and ICAM-1 antibody (BBA3) were purchased from R&D Systems (Minneapolis, MN, USA). Sheep anti-mouse IgG secondary antibody (NA931) was purchased from Amersham (Little Chalfont, Bucks, UK). 2,2′-azobis [2-methylpropioamidine] dihydrochloride (AAPH, 97%), fluorescein (puriss.p.a.) and (±)-6-hydroxy-2,5,7,8-tetramethylchromane-2-carboxylic acid (Trolox, 97%) were obtained from Sigma-Aldrich Chemie GmbH (Steinheim, Germany). Gallic acid, phenolic acids (syringic, vanillic, sinapic, benzoic, chlorogenic, gallic, p-coumaric, cinnamic, caffeic, ferulic, hydroxybenzoic and protocatechuic), 5-(hydroxymethyl)-2-furaldehyde (hydroxymethylfurfural; HMF) and Folin- Ciocalteu’s phenol reagent were purchased from Sigma-Aldrich (St Louis, MO, USA).

### Quantitative Analysis of Total Phenolics

Total phenolic content was determined by Folin-Ciocalteau method [22], where gallic acid was used as a calibration standard in photometric measurement performed by microplate reader (Varioskan Flash, Thermo Scientific, Finland).

### HPLC Analysis of Phenolic Acids and Hydroxymethylfurfural (HMF)

Ethyl acetate honey extracts in methanol solution were diluted fivefold with water, filtered (0.45 µm) and analyzed by HPLC (high-performance liquid chromatography) (Agilent 1100 Series HPLC-MSD, Agilent Technology) connected to a diode array detector (DAD) as described earlier [21]. Analyses were performed on a HyperClone ODS (C18) column (2.0 mm, 200 mm, 5 µm, Phenomenex) using the following gradient run (0.2 ml/min) with methanol (A) and 0.3% formic acid (B): 90–75% B from 0 to 5 min, 75–69% B from 5 to 20 min and 69–40% B from 20 to 40 min. The run was stopped at 65 min and post time was 25 min. Phenolic acids were quantified at 260 nm or 320 nm, and 5- (hydroxymethyl)-2-furaldehyde (HMF) at 280 nm. The authentic chemical compounds used for identification and quantification of phenolic acids were protocatechuic, benzoic vanillic, syringic, sinapic, trans-cinnamic, p-coumaric, caffeic, p-hydroxybenzoic, gallic, ferulic and chlorogenic acid.

### ORAC Assay

Oxygen radical absorbance capacity (ORAC) of the ethyl acetate honey extracts was analyzed by modified method of Huang et al. [23] as described in our earlier publication [24]. Ethyl acetate extracts in the methanol solutions were diluted (1000–3000 fold) in a phosphate buffer (75 mM, pH 7.4). Blank (methanol) and Trolox standards (15–100 µM) were also prepared in a phosphate buffer. Area under oxidation curve of each sample dilution was calculated and the ORAC values of ethyl acetate honey extracts were calculated using linear calibration curve of Trolox standard.

### Analysis of Sugars

Carbohydrates (glucose, fructose and sucrose) were analyzed by capillary electrophoresis (CE) modified from a method of Rovio et al. [25], with a P/ACE MDQ CE instrument (Beckman-Coulter, Fullerton, CA, USA) using diode array detection at 270 nm. Uncoated fused-silica capillaries of I.D. 25 µm and length 30/40 cm (effective/total length) were used. The ethyl acetate honey extracts were injected at a pressure of 0.5 psi for 10 s. The separation voltage was raised linearly within a 1 min ramp time from 0 to +16 kV. Calibration curves for the quantification of glucose, fructose and sucrose were prepared using standard solutions with concentrations between 10 and 430 mg/L.

### Cell Culture

Human aortic endothelial cells (HAEC), MCF-7 and PC-3 were obtained as cryopreserved cells from European Collection of Cell Cultures (ECACC) and were maintained in T-75 culture flasks at 37°C in a humidified 95% air-5% CO_2_ atmosphere. HAEC were grown in endothelial cell growth medium M200 (Cascade Biologics, Portland, OR) supplemented with 2% LSGS (low serum growth supplement, Cascade Biologics, Portland, OR) according to the manufacturer’s recommended protocol. Cells between passages 4–8 were used in this study. MCF-7 and PC-3 cells were maintained in Dulbecco’s minimal essential medium (DMEM) supplemented with 10% FBS, 100 U/ml penicillin, and 100 mg/ml streptomycin. Cells were cultured and were split according to standard procedures.

### Cell Viability and Cytotoxicity

Cell viability was determined using the colorimetric MTT metabolic activity assay [26]. MTT is cleaved in the mitochondria of metabolically active cells to form a colored, water-insoluble formazan salt. Briefly, breast and prostate cancer cells were grown in 96 flat-bottomed well plates and incubated for 48 hours with various concentrations of extracts (20–500 µg). As a positive control, MCF-7 and PC-3 cells were cultured with ICI 182780 and Doxorubicin respectively. Cells in their growth medium were used as control samples (vehicle). After the incubation, medium was replaced with MTT (1 mg/ml) dissolved in serum-free, phenol red-free medium, and incubation continued at 37°C for an additional 4 h. The MTT-formazan product was solubilized thoroughly in isopropanol and the optical density was measured at a test wavelength of 550 nm and a reference wavelength of 690 nm. The absorbance was used as a measurement of cell viability, normalized to cells incubated in control medium, which were considered 100% viable. Cell viability assay was also carried out for normal endothelial cells (HAEC) under the same experimental conditions.

### Cell ELISA

To measure the cell-surface expression of ICAM-1 and VCAM-1, cell ELISA was conducted. Confluent HAEC cultured in 96-well plates were pretreated with or without honey extracts for 18 h and then stimulated with 1 ng/ml TNF-α for 6 hours at 37°C. Subsequently, cells were fixed by 0.1% glutaraldehyde in PBS for 30 min at 4°C and plates were blocked at 37°C for 1 h with 5% skimmed milk powder in PBS. Monoclonal antibodies to ICAM-1 or VCAM-1 in 5% skimmed milk in PBS were then added to the wells and incubated at 4°C overnight. After washing away the excess unbound primary antibody, cells were further incubated with a horseradish peroxidase-conjugated sheep anti-mouse secondary antibody for 1.5 h at room temperature. The expression of cell adhesion molecules was quantified by the addition of the peroxidase substrate o-phenylenediamine hydrochloride. As a positive control we have used α-Tocotrienol (αΤ3, 25 µM), the most effective vitamin E analog in the reduction of cellular adhesion molecule expression and monocytic cell adherence [27]. The absorption of each well was measured at 450 nm using a microplate spectrophotometer.

### Statistical Analysis

Data is represented as mean±S.D. Statistical analysis was performed using Student’s t-test, two-tailed distribution, assuming two-sample unequal variance. Pearson’s correlation was carried out to identify relationships between phenolic acids and biological properties. The minimum level of significance was set at p<0.05.

## Results

### Phenolic Acid Composition of Honey Extracts

**Figure 1:**
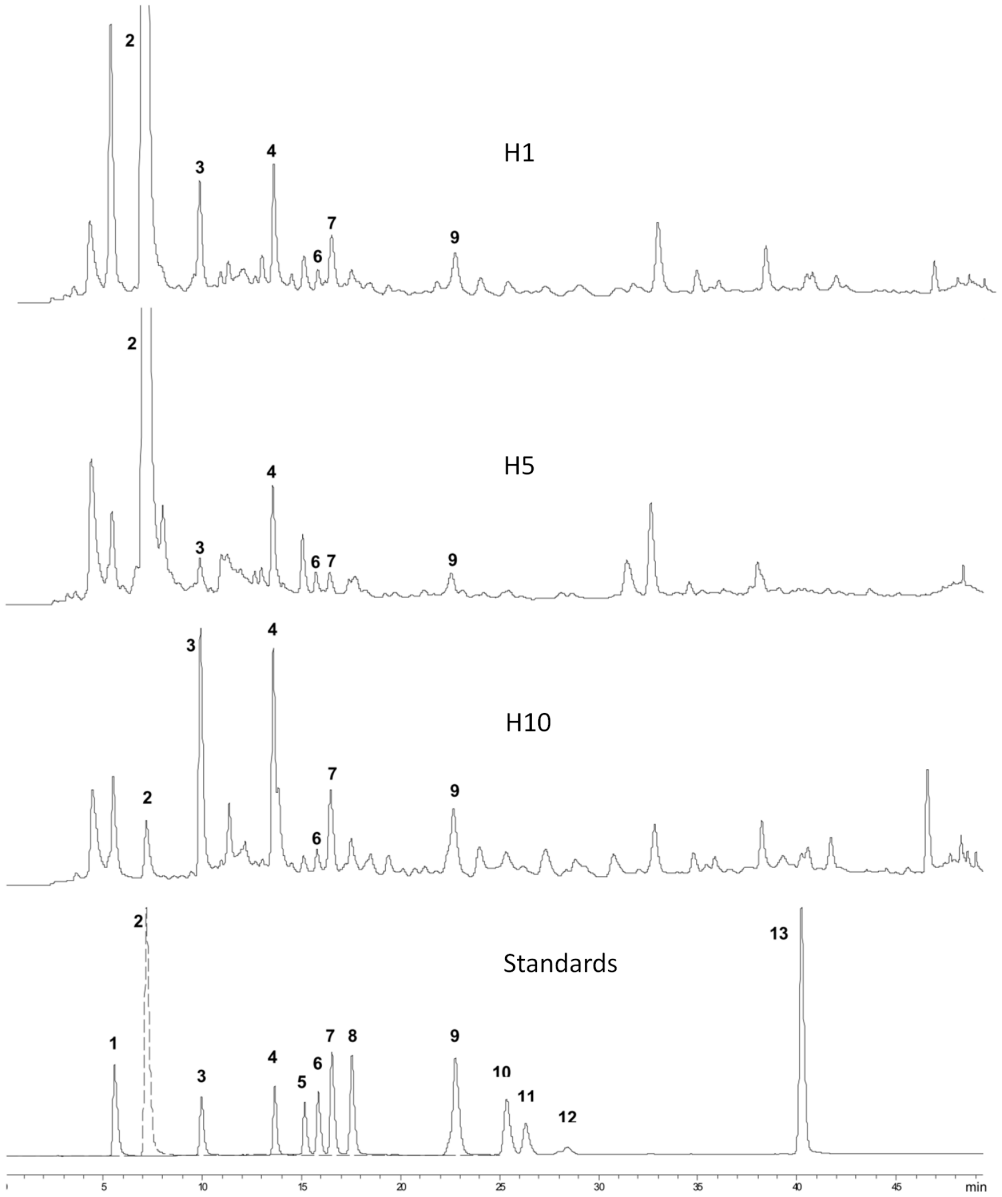
Representative chromatograms of honey extracts and standards. HPLC-DAD chromatograms (280 nm) obtained from honey samples (H1, H5, H10) and a standard mixture of phenolic acids and hydroxymethylfurfural (HMF). Peak identification: (1) gallic acid; (2) HMF; (3) protocatechuic acid; (4) p-hydroxybenzoic acid; (5) chlorogenic acid; (6) vanillic acid; (7) caffeic acid; (8) syringic acid; (9) p-coumaric acid; (10) ferrulic acid; (11) sinapic acid; (12) benzoic acid; (13) trans-cinnamic acid.

For the HPLC analysis of phenolic acids a total of 12 standards including protocatechuic, benzoic, vanillic, syringic, sinapic, trans-cinnamic, p-coumaric, caffeic, p-hydroxybenzoic, gallic, ferulic and chlorogenic acid were used. Examination of the HPLC chromatogram of honey ethyl acetate extracts revealed that Greek honeys are rich in phenolic acids. The phenolic acid pattern of the extracts was confirmed to contain protocatechuic, p-hydroxybenzoic, vanillic, caffeic and p-coumaric acid while gallic, ferulic, sinapic, syringic, trans-cinnamic and chlorogenic acid were not detected (Figure 1). The main constituent was protocatechuic acid. Protocatechuic and caffeic acid levels were higher in pine (mean values: 4513 and 558.5 µg/kg honey respectively) and fir honey (mean values: 8058.3 and 289.3 µg/kg honey respectively) when compared to thyme (mean value of protocatechuic acid: 471.3 µg/kg honey, mean value of caffeic acid: 122 µg/kg honey) or citrus honey (protocatechuic acid content: 303 µg/kg honey, caffeic acid content: 92 µg/kg honey). p-Hydroxybenzoic acid was the dominant constituent in thyme honeys (mean: 1252.5 µg/kg honey). In all samples, the amount of vanillic acid content ranged from 71 to 376 µg/kg honey and p-coumaric acid content ranged from 135 to 701 µg/kg honey (Table 2).

**Table 2:** Concentration of phenolic acids and hydroxymethylfurfural detected in honey ethyl acetate extracts^a^ ^,^ ^b^.

Floral source	PCA µg/kghoney	p-HBA µg/kghoney	VA µg/kghoney	CA µg/kghoney	p-COUA µg/kghoney	HMF mg/kghoney
Thyme Attiki (H1)	649±15	1070±25	225±6	134±3	146±4	7.5±0.2
Thyme 45% (H5)	346±25	1101±9	236±2	113±1	143±1	8.8±0.2
Thyme 55% (H11)	300±22	1724±79	245±11	121±5	252±11	11.1±0.5
Thyme 62% (H8)	590±21	1115±7	103±1	120±1	176±2	8.5±0.3
Fir (H9)	16777±780	1438±71	307±14	377±18	514±28	0.7±0.1
Fir (H7)	3258±24	1121±7	149±2	254±2	222±1	0.8±0.04
Fir Attiki (H4)	4140±131	1122±37	262±8	237±8	219±4	1.4±0.1
Pine (H10)	5967±57	4059±39	376±2	741±7	701±6	0.2±0.003
Pine (H6)	3058±111	1460±59	340±13	376±15	211±8	0.7±0.02
Forest Fino (H3)	2394±50	1503±41	237±7	445±12	288±11	3.0±0.1
Wild flowers-forest-thyme Attiki (H2)	1046±27	1124±25	201±5	255±5	193±5	6.1±0.2
Citrus (H12)	303±7	889±27	71±2	92±2	135±7	9.8±0.4

a PCA, protochatechuic acid, P-HBA, p-hydroxybenzoic acis, VA, vanillic acid, CA, caffeic acid, p-COUA, p-coumaric acid, HMF, hydroxymethylfurfural.

b All data expressed on a honey weight basis as means ± SD (n = 3 independent determinations).

### Total Phenolic Content, Hydroxymethylfurfural and Sugar Content of Honey Extracts

Hydroxymethylfurfural (HMF) content ranged between 0.2–3 mg/kg in conifer tree honey and between 7.5–11.1 mg/kg in thyme and citrus honey (Table 2). Total phenolic content of ethyl acetate extracts ranged from 11 to 52 mg of gallic acid/kg honey (Table 3). The mean glucose and fructose content was 112 and 481 mg/kg honey respectively. Sucrose was not detected in the extracts. It is important to mention that although sugars are expressed as mg/kg honey, they do not represent the real amount of glucose and fructose in honey samples. The values concern the concentration of sugars in the ethyl acetate extracts and are important because the biological tests were performed with these extracts.

**Table 3:** Glucose and fructose content, total phenolic content and ORAC values of honey ethyl acetate extracts^a^ ^,^ ^b^.

Floral source	Glucose mg/kg honey	Fructose mg/kg honey	TP (mg of GA/kg honey)	ORAC (µmol of TE/kg honey)
Thyme Attiki (H1)	90±9	308±57	17±0.3	421±45
Thyme 45% (H5)	97±5	279±27	18±0.2	415±29
Thyme 55% (H11)	117±2	311±5	24±0.9	692±93
Thyme 62% (H8)	124±2	325±15	18±0.6	469±69
Fir (H9)	109±4	319±16	52±0.2	2129±195
Fir (H7)	54±2	189±14	20±0.5	619±51
Fir Attiki (H4)	105±6	344±23	33±0.6	661±63
Pine (H10)	141±9	261±16	50±0.6	2068±314
Pine (H6)	75±1	144±3	11±0.2	712±107
Forest Fino (H3)	73±5	224±5	24±0.2	557±41
Wild flowers-forest-thyme Attiki (H2)	83±11	252±18	17±0.5	415±21
Citrus (H12)	112±6	481±18	14±0.4	455±46

a TP, total phenolic content, GA, gallic acid, TE, Trolox equivalent.

b All data expressed on a honey weight basis as means ± SD (n = 3 independent determinations).

### Antioxidant Activity of Honey Extracts

The antioxidant potential of the extracts was measured using the ORAC assay (Table 3). ORAC values, expressed as Trolox equivalent (TE)/kg honey ranged from 619 to 2129 µmol TE/kg for pine and fir tree honeys and from 415 to 692 µmol TE/kg for thyme and citrus honeys indicating a higher antioxidant capacity for conifer tree honeys.

### Effect of Honey Extracts on ICAM-1 and VCAM-1 Expression

**Figure 2:**
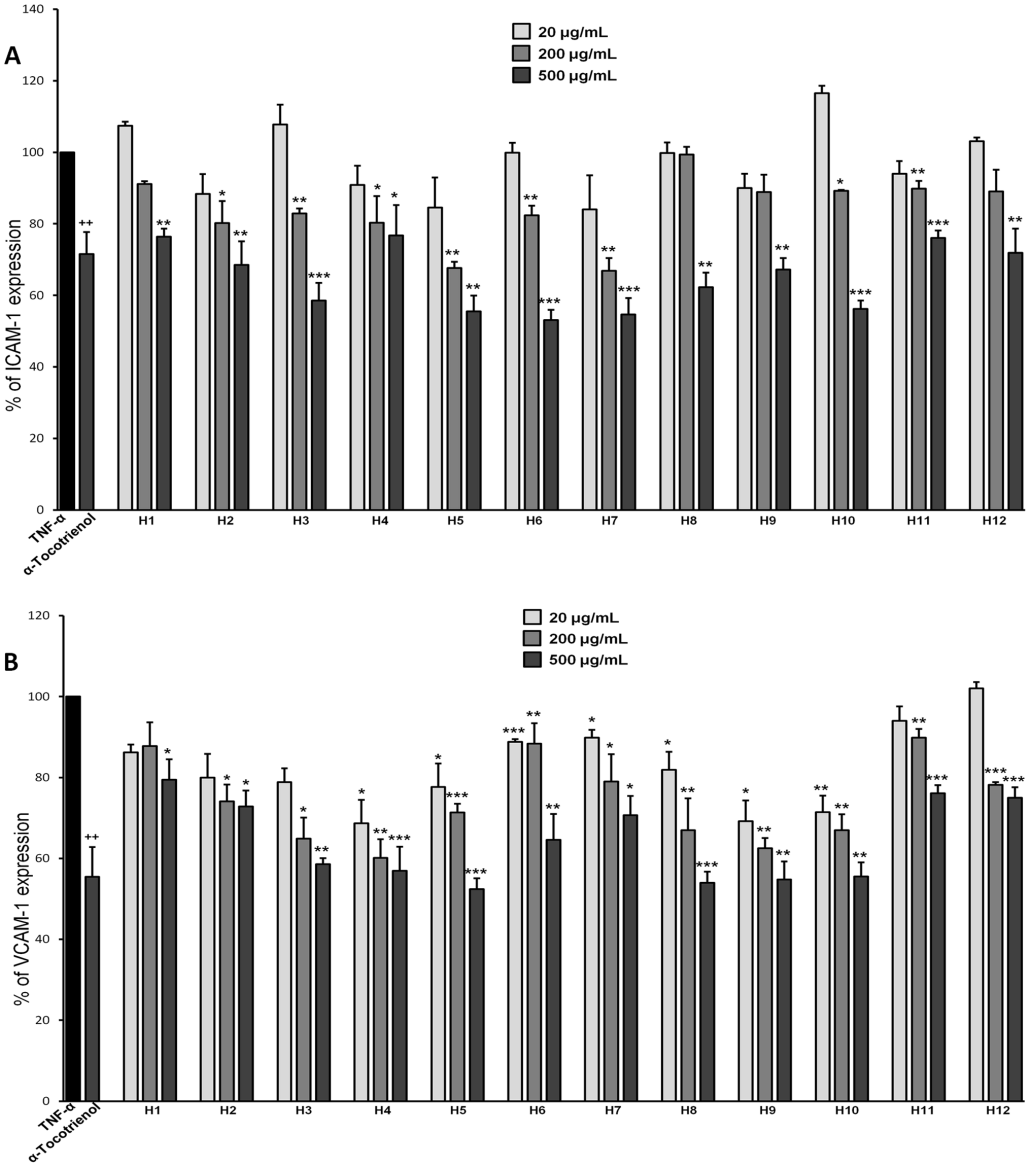
Greek honey extracts inhibit TNF-α-induced adhesion molecule expression. Greek honey extracts (H1–H12) inhibit TNF-α-induced ICAM-1 (A) and VCAM-1 (B) protein expression in HAEC. HAEC were incubated in the absence of TNF-α or compounds (control), with αT3 (25 µΜ), or with different concentrations (20–500 µg/ml) of honey extracts for 18 h, followed by stimulation with TNF-α (1 ng/mL) for up to 24 h. Adhesion molecules were measured by cell ELISA. Results are expressed as percent of control. A *p<0.05 value was considered statistically significant when compared to TNF-α-treated cells (**p<0.01, ***p<0.001). Values represent mean ± SD based on three independent experiments performed in triplicate.

TNF-α (1 ng/ml), increased the basal expression of ICAM-1 and VCAM-1 in HAEC. α-Tocotrienol decreased significantly the TNF-α-induced endothelial expression of both ICAM-1 and VCAM-1 as expected. Pretreatment of HAEC with different concentrations of honey extracts (20–500 µg/ml) for 18 h followed by TNF-α stimulation for another 6 hours inhibited significantly ICAM-1 and VCAM-1 cell surface expression (Figure 2A and 2B respectively). Conifer tree honeys (fir honey Attiki, fir honey from Karpenisi and pine honey from Chalkidiki) caused a significant VCAM-1 inhibition (30%) at a low concentration (20 µg extract/ml) whereas VCAM-1 inhibition was higher (40%) at a concentration range 200–500 µg extract/ml. Other honeys (thyme honeys from Crete containing 45% and 62% of pollen grains and forest honey Fino) inhibited significantly VCAM-1 expression (30%) only at higher concentrations (200–500 µg extract/ml). Incubation with 20–200 µg/ml of honey extracts did not result in endothelial cell cytotoxicity. Incubation with 500 µg/ml of the extracts led to a 7–12% inhibition of endothelial cell viability (data not shown); however, this percentage inhibition is considerably lower than the magnitude of the effect of extracts on adhesion molecule expression at this concentration.

### Effect of Honey Extracts on Viability of Cancer Cells

**Figure 3:**
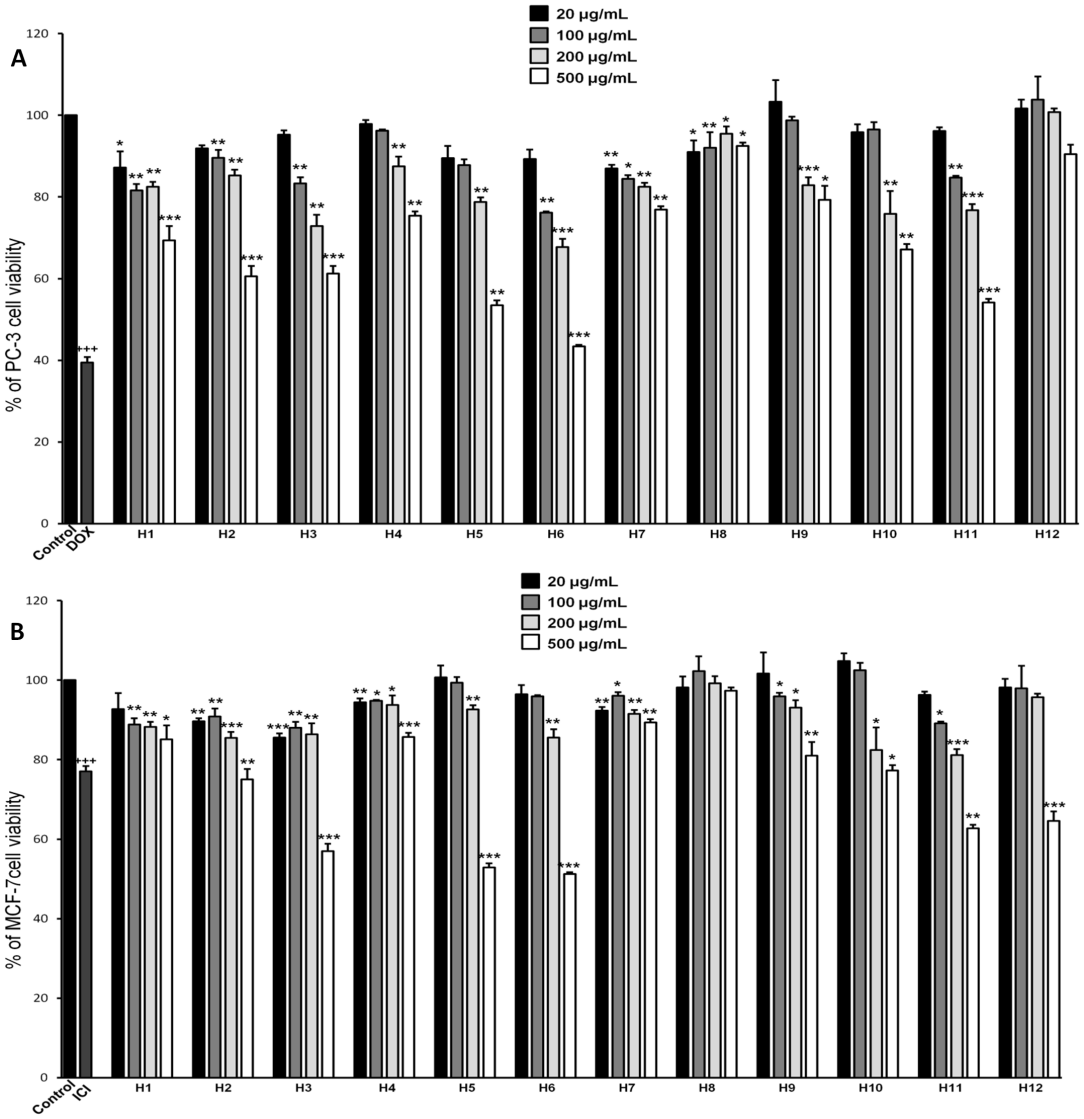
Greek honey extracts inhibit viability of prostate and breast cancer cells. Greek honey extracts (H1–H12) inhibit viability of PC-3 (A) and MCF-7 (B) cells. Cells were incubated in the absence of compounds (control) or with different concentrations (20–500 µg/ml) of honey extracts for 48 h. As a positive control, MCF-7 and PC-3 cells were cultured with ICI 182780 (0.1 µΜ) and doxorubicin (1 µΜ) respectively. After treatment, cells were subjected to the MTT assay. Results are expressed as percent of control. A *p<0.05 value was considered statistically significant when compared to TNF-α-treated cells (**p<0.01, ***p<0.001). Values represent mean ± SD based on three independent experiments performed in triplicate.

The inhibitory effect of honey extracts on the viability of PC-3 and MCF-7 cells is shown in Figure 3A and 3B respectively. Most honeys i.e. conifer tree honey as well as thyme honey, caused a significant PC-3 viability inhibition (30–60%) at a high concentration (500 µg extract/ml) whereas at a lower concentration (200 µg extract/ml) only four honeys (pine honey from Euboea, forest honey Fino, pine honey from Chalkidiki and thyme honey from Crete containing 55% of pollen grains) caused a significant cell viability inhibition (25–30%). Similarly, most honeys significantly reduced MCF-7 cell viability (20–50% inhibition) at a high concentration (500 µg extract/ml). Four honeys (pine honey from Euboea, forest honey Fino, wildflowers/forest/thyme honey Attiki and thyme honey from Crete containing 55% of pollen grains) caused a significant MCF-7 cell viability inhibition (15%, p<0.01) at a lower concentration (200 µg extract/ml).

### Association between Phenolic Acid Content and Biological Activities

As shown in Table 4, there were significant correlations between the antioxidant activity of honeys and protocatechuic acid content (r: 0.8497, p<0.001), p-hydroxybenzoic acid (r: 0.7079, p<0.01), vanillic acid (r: 0.6426, p<0.05), caffeic acid (r: 0.7315, p<0.01), p-coumaric acid (r: 0.9478, p<0.001) as well as with the total phenolic content of honeys (r: 0.926, p<0.001). There was also a significant correlation between VCAM-1 expression and total phenolic content (r: −0.6979, p<0.01) besides protocatechuic and vanillic acid content of honeys (r: −0.5749, p<0.05 and r: −0.5747, p<0.05 respectively). Vanillic acid content correlated significantly with MCF-7 and PC-3 cell viability inhibition (r: 0.6239, p<0.05, r: 0.7867, p<0.01 respectively).

**Table 4:** Correlation study between the results of biological tests and phenolic acid content^a^ ^,^ ^b^ ^,^ ^c^.

	PCA	p-HBA	VA	CA	p-COUA	TP	ORAC
ORAC	0.8497 (***)	0.7079 (**)	0.6426 (*)	0.7315 (**)	0.9478 (***)	0.926 (***)	1
VCAM-1 expression	−0.5749 (*)	−0.3482	−0.5747 (*)	−0.5174	−0.5493	−0.6979 (**)	−0.5357
PC-3 inhibition	0.1331	0.4126	0.7867 (**)	0.5355	0.3255	0.1283	0.2323
MCF-7 inhibition	−0.0695	0.5620	0.6239 (*)	0.4888	0.3694	0.1322	0.2048

a PCA, protochatechuic acid, p-HBA, p-hydroxybenzoic acid, VA, vanillic acid, CA, caffeic acid, p-COUA, p-coumaric acid, TP, total phenolic content.

b Values represent Pearson’s correlation coefficient (r).

c The minimum level of significance was set at p<0.05 (*), p<0.01 (**), p<0.001 (***).

## Discussion

Honey’s beneficial health effects result from its active constituents including flavonoids and phenolic acids. Various honeys have been analyzed regarding their phenolic acid content which is rather variable and depends mainly on the floral and geographical origin of honey. In this study, we investigated the phenolic acid profile as well as the antioxidant, anticancer and antiinflammatory/antiatherogenic properties of twelve ethyl acetate extracts derived from Greek honeys, collected from different regions in Greece.

Different extraction methods or solvents yield different concentrations of phenolic compounds. In our study we have used ethyl acetate extracts because ethyl acetate results in higher recovery of phenolic acids than diethyl ether and ethyl acetate extracts seem to possess higher biological activity compared to other extracts [4], [28].

HPLC analysis indicated that Greek honey ethyl acetate extracts contain high amounts of phenolic acids including protocatechuic acid, p-hydroxybenzoic acid, vanillic acid, caffeic acid and p-coumaric acid. The most abundant phenolic acid in this study was found to be protocatechuic acid. To our knowledge, protocatechuic acid is not detectable in most of the examined honeys derived from other countries, not even in “active Manuka honey”, a well-known for its antibacterial properties honey from New Zealand which has been used as a gold standard for comparison with other honeys. Diethyl ether extracts from Turkish honeydew honeys only (pine and oak) have been shown to contain protocatechuic acid in the range of 1639 to 5986 µg/kg honey [15]. The presence of protocatechuic acid in all Greek honeys implicates its potential to be used as a characteristic indicator of the origin of honey. The concentrations of protocatechuic acid ranged from 3058 to 5967 µg/kg honey for pine honeys while in fir honeys the concentration ranged from 3258 to 16777 µg/kg honey. The content of protocatechuic acid in Greek thyme honey (300–649 µg/kg honey) and citrus honey (303 µg/kg honey) was considerably lower than in conifer honeys. This variation might be attributed to the botanical origin of honey. In agreement with our results, Haroun et al. (2012) reported the presence of protocatechuic acid in conifer tree honeys but not in honeys from other floral sources, supporting that protocatechuic acid may be used for the differentiation of conifer tree honey from floral honeys [15]. The mean vanillic, caffeic and p-coumaric acid levels (229, 272.1 and 266.67 µg/kg honey respectively) in Greek honey extracts were similar to those reported for ethyl acetate or ether extracts from honeys derived from other geographical regions [4], [15], [29]. However, the mean caffeic acid levels in conifer honeys were significantly higher than thyme and citrus honeys. p-Hydroxybenzoic acid was the second most abundant phenolic acid (after protocatechuic acid) in Greek conifer honey extracts (mean: 1840 µg/kg honey), but it was the dominant phenolic acid in Greek thyme honeys (mean: 1252.5 µg/kg honey). Quantitative analysis of phenolic acids in ethyl acetate or ether extracts from Turkish, Cuban and Malaysian honeys revealed that p-hydroxybenzoic acid was not among the phenolic acids detected [4], [15], [29]. Our data indicate that the phenolic acid composition of Greek honeys differs significantly than that of honeys originated from other countries, implicating that geography influences the honey phenolic acid profile.

Honey supplementation has been reported to be beneficial in diabetes mellitus [30]. Evidence suggests that protocatechuic and caffeic acid are potent antidiabetic compounds [19], [20], which implicates that protocatechuic acid and caffeic acid may significantly contribute to the antidiabetic properties of honey. The current study reveals the unique composition of Greek conifer honey in protocatechuic acid, thus highlighting its utility as a potential antidiabetic agent.

In order to decide whether a physiologically achievable serving of honey (2–3 teaspoons) would result in biologically active protocatechuic acid levels we considered it important to take into account the following: 1) one teaspoon corresponds approximately to 20 g of honey, 2) our quantitative analysis data indicate that 50 g (2–3 teaspoons) of Greek conifer honey may contain 0.15–0.84 mg protocatechuic acid, 3) a bioavailability study in humans revealed that consumption of 350 ml wine containing 0.56 mg protocatechuic acid resulted in plasma concentration 0.2 µΜ, 4 hours after ingestion [31], 4) a recent study in mice supports that protocatechuic acid is a potent antidiabetic agent when plasma protocatechuic acid levels range from 0.06 to 0.13 µΜ. Importantly, protocatechuic acid administration to mice has been shown to increase its deposit in plasma and organs [19]. Taken together, we suggest that a daily intake of 2–3 teaspoons of conifer honey may result in plasma protocatechuic acid concentrations with potential antidiabetic activity. However, clinical studies are necessary to evaluate the utility of conifer honey in diabetes.

For the determination of the antioxidant activity of honey extracts we used the ORAC assay, which measures peroxyl radical scavenging capacity [23] and is widely used in food sector. The ORAC values of the extracts ranged between 415 and 2129 µmol TE/kg honey and were higher in conifer honeys (mean: 1237.8 µmol TE/kg honey) than thyme and citrus honeys (mean: 490.4 µmol TE/kg honey). The use of different honey fractions i.e. total honey, water soluble or organic solvent fractions have provided a wide range of antioxidant activity values. Comparison of our data with ORAC values from studies using similar fractions revealed that Greek honey possess high antioxidant potential; for example, ether extracted methanol fractions of native monofloral Cuban honeys varied from 430 to 1220 µmol TE/kg honey while in another study, the ORAC values for ether extracts of various monofloral commercial honeys from North America ranged from 110 to 900 µmol TE/kg honey [5], [29].

Accumulating evidence indicates that honey is cardiopotective and vasoactive *in vivo* [32] while *in vitro* experiments demonstrate honey’s protective activity in a cultured endothelial cell line subjected to oxidative stress [10]. However, the effect of honey on the inflammatory process of atherosclerosis remains unknown. In the present study, we have evaluated the antiinflammatory action of ethyl acetate honey extracts, by assessing their potential to inhibit TNF-α-induced activation of endothelial cells. For this purpose, cell ELISA, a widely used *in vitro* assay measuring the antiinflammatory effects of examined compounds or extracts in endothelial cells was conducted. As a positive control we have used α-Tocotrienol, a novel vitamin E with superior activity over tocopherol with regard to the attenuation of the expression of adhesion molecules. Incubation with Greek honey extracts caused a significant reducing effect on TNF-α-activated adhesion molecule expression. It is important to say that conifer tree honeys showed the highest inhibitory effect. These data indicate the potential of Greek honeys to inhibit the initial step of atherosclerotic process.

Honey has been previously shown to protect against various types of cancer, like bladder, colon and breast cancer [2], [6], [7], [33], [34]. In the present study, we demonstrate that Greek honey extracts caused cell death of breast and prostate cancer cell lines. Particularly, the ethyl acetate honey extracts inhibited significantly MCF-7 and PC-3 cell viability at concentrations ranging from 200 to 500 µg of extract/ml which correspond to 20–50% w/v of entire honey. Manuka honey has also been shown to inhibit significantly MCF-7 cell viability when an aqueous solution 2.5–5% w/v of entire honey was added to the cells [6]. Similarly, native Tualang and Indian honey when added directly to cells inhibited MCF-7 cell viability showing IC_50_ values of 2.4 and 4% respectively [7], [34]. In another study, honey inhibited proliferation of PC-3 cells and IC_50_ concentration was 2.4% [35]. The anticancer activity shown by our ethyl acetate extracts is not comparable to the activity demonstrated by the entire honey because entire honey is a supersaturated solution of sugars and contains more active substances such as other phenolic compounds embedded in sugars and terpenes [1], [10].

Given that phenolic acids are considered strong antioxidants and known to inhibit cancer related pathways, we further looked for features that could account for the differences in the biological activities between the various honey extracts. A significant correlation was found between vanillic acid content and inhibition of breast and prostate cancer cell viability. This suggests that vanillic acid may be the major component responsible for the anticancer activity observed. Vanillic acid has been previously shown to be an effective anticancer agent in vitro [36]. Each of the single phenolic acids present in the ethyl acetate extracts (protocatechuic acid, p-hydroxybenzoic acid, vanillic acid, caffeic acid and coumaric acid) as well as the total phenolic content, were highly correlated with the ORAC values. Thus, antioxidant property of the extracts can be attributed, at least in part, to the presence of these phenolic acids. The inhibitory effect of honey extracts on TNF-α-induced VCAM-1 was also significantly correlated with the total phenolic content as well as with the protocatechuic and vanillic acid content. This is consistent with the fact that antioxidant agents including protocatechuic aldehyde are inhibitors of TNF-α-inducible VCAM-1 expression in endothelial cells [37].

In conclusion, our findings suggest that Greek honeys are particularly rich in phenolic acids and exhibit significant antioxidant, anticancer and antiatherogenic activity. Greek conifer tree honeys are a rich source of protocatechuic and caffeic acid, implicating their beneficial use in patients with diabetes mellitus.

## Supporting Information

Table S1 **Plant species recorded in honey samples through microscopic examination.** Pollen grains of *Thymus capitatus* were found in four honeys in the range of 35% to 62%. Six conifer honeys (fir and pine), one honey comprised of a mixture of wildflowers, forest and thyme and one honey from citrus were also characterized.
(DOCX)
