# Supplementary material for: Phenolic Acid Composition, Antiatherogenic and Anticancer Potential of Honeys Derived from Various Regions in Greece
Source: PLoS One. 2014 Apr 21;9(4):e94860. doi: 10.1371/journal.pone.0094860 (PMC3994057; doi:10.1371/journal.pone.0094860)
Supplement: Table S1 — Plant species recorded in honey samples through microscopic examination. Pollen grains of Thymus capitatus were found in four honeys in the range of 35% to 62%. Six conifer honeys (fir and pine), one honey comprised of a mixture of wildflowers, forest and thyme and one honey from citrus were also characterized. (DOCX) [file pone.0094860.s001.docx]

**Table S1** Plant species recorded in honey samples. The numbers illustrate percentages (%) of pollen grain frequencies.

|  | Thyme Attiki (H1) | Thyme 45% (H5) | Thyme 55% (H11) | Thyme 62% (H8) | Fir (H9) | Fir (H7) | Fir Attiki (H4) | Pine (H10) | Pine (H6) | Forest Fino (H3) | Wild flowers-forest-thyme Attiki (H2) | Citrus (H12) |
| --- | --- | --- | --- | --- | --- | --- | --- | --- | --- | --- | --- | --- |
| Taxa |  |  |  |  |  |  |  |  |  |  |  |  |
| **Boraginaceae** |  |  |  |  |  |  | 7 |  |  |  |  |  |
| *Echium* sp. |  | 2 |  |  |  |  |  |  |  |  |  |  |
| **Chenopodiaceae** |  |  |  |  |  |  |  |  |  |  |  |  |
| *Chenopodium* sp. |  |  |  | √ |  |  | √ |  |  |  |  |  |
| **Cistaceae** |  |  |  |  |  |  |  |  |  |  |  |  |
| *Cistus* sp*.* | √ | √ | √ | √ | √ | √ | √ | √ |  | √ | √ |  |
| *Helianthemum* sp. |  |  | √ |  |  |  |  |  |  |  |  |  |
| **Compositae** | 1 | 5 |  | 3 |  | 5 | 4 | 1 | 2 | 3 | 2 | 7 |
| *Centaurea* sp. |  |  |  |  |  |  |  | <1 |  |  |  |  |
| **Convolvulaceae** |  |  |  |  |  |  |  |  |  |  |  |  |
| *Convolvulus* sp. |  |  |  | √ |  |  |  | √ |  |  |  |  |
| **Cruciferae** |  |  |  |  |  |  |  |  |  |  |  |  |
| *Brassica* sp. |  | 5 | 3 | 1 |  | 40 |  |  |  |  | 6 | 45 |
| **Ephedraceae** |  |  |  |  |  |  |  |  |  |  |  |  |
| *Ephedra* sp. |  |  |  |  |  |  |  |  |  |  | √ |  |
| **Ericaceae** |  |  |  |  |  |  |  |  |  |  |  |  |
| *Erica* sp. | 12 |  | 8 |  |  |  | 55 | 4 | 31 | 25 | 6 | 25 |
| **Fagaceae** |  |  |  |  |  |  |  |  |  |  |  |  |
| *Castanea sativa* Miller |  |  | <1 |  | 85 |  |  |  |  | 10 | 17 |  |
| *Quercus ilex* L. |  |  | √ |  |  | √ |  |  |  |  |  |  |
| **Labiatae** |  |  |  |  |  |  |  |  |  |  |  |  |
| *Ballota* sp. | 1 | 14 | 15 |  |  |  |  |  |  |  |  |  |
| *Phlomis* sp. | 3 | 2 | 3 | 2 |  |  | 1 |  |  |  | 1 |  |
| *Thymus capitatus* (L.) Hoffmans & Link*.* | 35 | 45 | 55 | 62 |  |  |  |  |  |  | 6 |  |
| **Leguminosae** |  | 2 |  |  |  |  |  |  |  |  | 26 |  |
| *Trifolium* sp. | 18 | 6 |  | 8 | 10 |  | 3 | 6 | 4 | 14 | 6 |  |
| *Vicia* sp. |  |  |  |  |  | 5 |  | <1 |  |  |  |  |
| **Liliaceae** |  |  |  |  |  |  | 2 |  | 2 |  | 1 | 12 |
| **Myrtaceae** |  |  |  | 15 |  |  |  |  |  |  |  |  |
| *Eucalyptus camaldulensis* Dehnh. |  | 8 | 6 | 5 |  |  |  |  |  | 1 | 1 |  |
| *Myrtus communis* L. | 23 | 8 | 10 | 7 |  |  |  |  |  |  | <1 |  |
| **Oleaceae** |  |  |  |  |  |  |  |  |  |  |  |  |
| *Olea europaea* L. | √ | √ | √ | √ |  |  |  |  |  |  | √ | √ |
| **Oxalidaceae** |  |  |  |  |  |  |  |  |  |  |  |  |
| *Oxalis* sp. |  |  |  |  |  |  |  |  |  |  |  | 7 |
| **Polygonaceae** |  |  |  |  |  |  |  |  |  |  |  |  |
| *Polygonum aviculare* L. |  |  |  |  |  |  |  | 31 |  | 29 | 4 |  |
| **Rhamnaceae** |  |  |  |  |  |  |  |  |  |  |  |  |
| *Paliurus spina-christi* Miller |  |  |  |  |  | 5 |  |  |  |  | 2 |  |
| **Rosaceae** |  |  |  |  |  |  | 5 |  |  |  |  |  |
| *Rubus* sp. | 1 |  | <1 |  |  |  |  |  |  |  | <1 |  |
| **Rutaceae** |  |  |  |  |  |  |  |  |  |  |  |  |
| *Citrus* sp. |  |  |  |  |  |  |  |  |  |  |  | 1 |
| **Smilacaceae** |  |  |  |  |  |  |  |  |  |  |  |  |
| *Smilax* sp. |  |  |  |  |  |  |  |  | 5 | 1 |  |  |
| **Umbelliferae** | 4 | 2 |  |  |  | 12 |  |  |  |  | 6 |  |
| *Bubleurum* sp. |  |  |  | 1 |  |  |  | 3 |  |  |  |  |
|  |  |  |  |  |  |  |  |  |  |  |  |  |
| **HDE/PG** |  |  |  |  |  |  |  | 1,25 | 1,01 | 0,19 |  |  |

**√** indicates nectarless plant species

**HDE/PG** indicates ratio of honeydew elements to pollen grains
